# Association of blood cadmium concentration with chronic obstructive pulmonary disease progression: a prospective cohort study

**DOI:** 10.1186/s12931-024-02726-0

**Published:** 2024-02-17

**Authors:** Jing Sun, You-Peng Deng, Juan Xu, Feng-Min Zhu, Qi-Yuan He, Min-Min Tang, Ying Liu, Jin Yang, Hong-Yan Liu, Lin Fu, Hui Zhao

**Affiliations:** 1 Department of Respiratory and Critical Care Medicine, The Second Affiliated Hospital of Anhui Medical University, Hefei, 230601 Anhui China; 2 Institute of Respiratory Diseases, The Second Affiliated Hospital of Anhui Medical University, Hefei, 230601 Anhui China; 3 Center for Big Data and Population Health of IHM, The Second Affiliated Hospital of Anhui Medical University, Hefei, 230601 Anhui China

**Keywords:** Chronic obstructive pulmonary disease, Cadmium, Acute exacerbation, Death, Progression, Cohort study

## Abstract

**Background:**

Prior studies in patients with chronic obstructive pulmonary disease (COPD) had indicated a potential correlation between cadmium (Cd) exposure and reduction in lung function. Nevertheless, the influence of Cd exposure on the progression of COPD remained unknown. Exploring the relationship between Cd exposure and the progression of COPD was the aim of this investigation.

**Methods:**

Stable COPD patients were enrolled. Blood samples were collected and lung function was evaluated. Regular professional follow-ups were conducted through telephone communications, outpatient services, and patients' hospitalization records.

**Results:**

Each additional unit of blood Cd was associated with upward trend in acute exacerbation, hospitalization, longer hospital stay, and death within 2 years. Even after adjusting for potential confounding factors, each 1 unit rise in blood Cd still correlated with a rise in the frequencies of acute exacerbation, longer hospital stay, and death. Moreover, COPD patients with less smoking amount, lower lung function and without comorbidities were more vulnerable to Cd-induced disease deterioration.

**Conclusion:**

Patients with COPD who have higher blood Cd concentration are susceptible to worse disease progression.

**Graphical Abstract:**

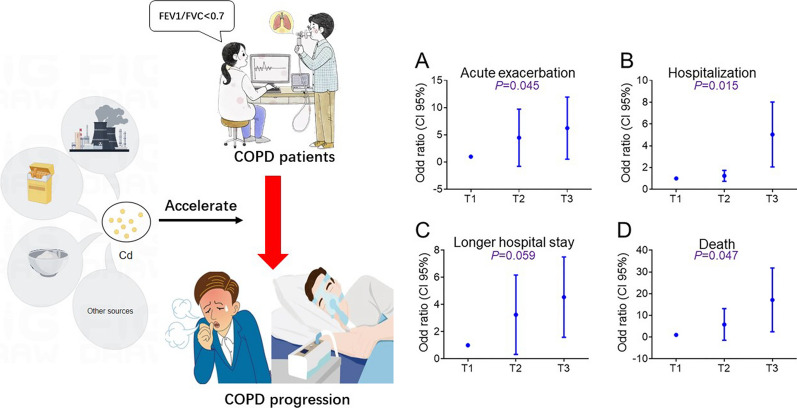

## Introduction

Chronic obstructive pulmonary disease (COPD) is characterized by ongoing respiratory difficulties, restricted airflow, and a variety of general symptoms [22, 41]. This disease is prevalent globally and poses substantial financial and medical burdens, with projections indicating a further increase in the near future [23]. During COPD acute exacerbation, patient's respiratory symptoms, including dyspnea, increased mucus production, and coughing worsen beyond their usual levels, requiring modifications in their prescriptions and are associated with a higher mortality risk [1, 30, 31]. The prevention of COPD exacerbation is crucial, as exacerbation has adverse and long-term effects on patient's health, leading to cumulative lung function decline [17, 35, 36]. The progression of COPD can be influenced by both infectious and noninfectious reasons. Noninfectious causes such as airborne contaminants, toxic substances, and comorbidities play an important role [21]. A greater emphasis has been placed in recent years on noninfectious factors, such as heavy metals. Nevertheless, it is unclear whether exposure to heavy metals affects COPD progression.

Cadmium (Cd) is an extensively prevalent occupational and environmental heavy metal detected in many sources such as food, air pollution, occupational dust, and smoking [4, 10, 18, 38, 43]. Cd has a remarkably long half-life in the body, existing over approximately 25–30 years in mammals [9, 20]. Inhaling Cd-laden smoke can lead to acute lung injury, diffuse alveolar injury, lung cancer, pulmonary fibrosis, emphysema, and chronic pulmonary inflammation [13, 15, 26, 29]. A cross-sectional study has shown a high correlation between increased blood Cd level and decreased lung function in COPD patients, indicating potential pulmonary toxicity of Cd [47]. However, the exact correlation between Cd and the progression of COPD is unclear at this time.

The cohort research was conducted to explore the correlation between exposure to Cd and the progression of COPD. The study comprised COPD patients from three tertiary hospitals in Anhui Province, whose blood Cd concentrations were evaluated and the progressions were followed up.

## Materials and methods

### Patients

The Anhui COPD cohort (AHCC) was used to select patients with COPD for this investigation [32, 47]. In Anhui, the investigation was carried out at three tertiary hospitals, named the Second Affiliated Hospital of Anhui Medical University, Bozhou People's Hospital, and Fuyang People's Hospital. Each subject was a patient from one of these hospitals' Department of Respiratory and Critical Care Medicine. To ensure similarity in lifestyles and environmental circumstances, only COPD patients who had resided in Fuyang, Hefei, or Bozhou for five years or more were included in the study after preliminary screening. 196 COPD patients in all were enrolled between September 2020 and June 2022 and they all satisfied with the criteria of inclusion. The following were the study’s inclusion requirements: 1. According to the American Thoracic Society and Global Chronic Obstructive Pulmonary Disease (GOLD) standards, patients were given a COPD diagnosis [40], 2. Willingness to participate in subsequent follow-ups; 3. Willingness to cooperate with the investigation and undergo peripheral blood collection and lung function test. The exclusion criteria included: 1. Incomplete information and inability to participate in follow-ups; 2. Presence of malignant tumors or autoimmune disorders; 3. Coexistence of bronchiectasis, pneumonia, or other respiratory diseases; 4. Unacceptable blood samples. All participants provided blood samples, completed questionnaires, underwent lung function examinations, and were followed up annually. Researchers also gathered information on drug use, alcohol consumption, and smoking habits through questionnaires.

### Data and sample collection

The hospitals' electronic medical record systems were used to collect clinical characteristics, blood test results, and demographic information. Prior to venous blood collection, patients were instructed to fast for at least six hours. Within two hours of collection, samples were quickly centrifuged at 3000 RPM for 10 min. The blood specimens were pre-thawed in a freezer set at 4 °C and isolated before analysis [9].

### Blood Cd detection

The baseline level of blood Cd was detected through inductively coupled plasma mass spectrometry (ICP-MS, Nex-ION350X, PerkinElmer, Shelton, CT 06484, USA) [24]. The whole blood samples (400 μL) were diluted with dilution (180 μL: 3820 μL). The blood Cd detection limit was 0.001 ppm. According to the tertiles of blood Cd concentration, the low Cd group (Tertile 1: Cd < 0.367 ppm), the medium Cd group (Tertile 2: Cd: 0.367 ~ 1.102 ppm), and the high Cd group (Tertile 3: Cd > 1.102 ppm) were established among the whole participants.

### Progress follow-up

Regular follow-ups were conducted primarily through phone communications, outpatient visits, and patients' hospitalization records. The follow-up process was carried out by a team consisting of doctors specializing in respiratory illness, all of whom had undergone comprehensive training and held valid medical certificates. The follow-up materials used in this study were professionally designed questionnaires. Since joining the group, COPD patients were followed up once a year, and the resulting indices of disease progression were averaged within 2 years. Depending on the frequency of acute exacerbation in the 75th percentile quantile, subjects were split into two groups: a low acute exacerbation group (≤ 2), and a high acute exacerbation group (> 2). According to the 75th percentile quantile of the total frequency of hospitalization, the subjects were split into two groups: one with low hospitalization (≤ 2), and the other with high hospitalization (> 2). The subjects were divided into three subgroups according to the tertiles of the length of hospital stay: tertile 1: ≤ 8 days; tertile 2: 8 ~ 15 days; tertile 3: ≥ 15 days. In the stratified analysis, age, smoking amount, forced vital capacity (FVC), forced expiratory volume in one second (FEV1), FEV1/FVC%, and predicted FEV1% were divided into two groups according to the 75th percentile.

### Statistical analysis

The expression for continuous variables were mean (standard error) and median (25th, 75th), respectively. The percentages of numbers were used to represent categorical variables. The Kruskal–Wallis nonparametric analysis of variance was used to examine differences between several groups. The Bonferroni correction was used to adjust P-values for multiple comparisons. The difference between the two groups was contrasted using the Mann–Whitney U test or chi-square test. Before conducting logistic models, collinearity diagnostics were used to check for any potential overlap between independent variables. To evaluate the prediction effectiveness of factors and calculate odds ratios (OR) and 95% confidence intervals (CI), logistic regression and linear regression models were used in both univariate and multivariate studies. Age, sex, smoking status, smoking amount, comorbidities, inhaled therapy for COPD, and pulmonary function indices were adjusted. Stratified analysis was conducted by smoking amount, age, sex, FVC, FEV1, FEV1/FVC%, predicted FEV1%, hypertension, diabetes mellitus, coronary disease, and cerebrovascular diseases. A P-value of 0.05 or less was considered statistically significant.

## Results

### Demographic characteristics

There were 196 eligible COPD patients in the study. Table 1 presented their demographic characteristics and clinical information. All participants were 74.1 years old on average, with 146 (74.5%) were male, 33 (16.8%) were current smoking, 77 (39.3%) were former smokers, and 86 (43.9%) had never smoked. Regarding comorbidities, 92 (46.9%) patients had hypertension, 21 (10.7%) had diabetes, 25 (12.8%) had coronary disease, and 23 (11.7%) had cerebrovascular disease. Comparing individuals with COPD in different blood Cd groups, no differences in sex, age, comorbidities, or inhaled therapy were observed. It was worth noting that the number of cigarettes smoked did increase as blood Cd concentration rose. Additionally, certain clinical indicators, including C-reactive protein (CRP), interleukin-6 (IL-6), white blood cells (WBCs), neutrophils, monocytes, and alanine aminotransferase (ALT) were markedly raised in parallel with blood Cd level. On the other hand, there were no notable differences between the various blood Cd groups in assays for uric acid, urea nitrogen, lymphocytes, eosinophils, basophils, aspartate aminotransferase (AST), creatinine, and estimated glomerular filtration rate (eGFR). Importantly, as blood Cd level increased, FEV1, FVC, FEV1/FVC%, and predicted FEV1% decreased considerably (Table 1).

**Table 1:** Demographic characteristics of participators at baseline

Characteristic	All participators	Tertile of blood Cd			*P*
		Tertile 1 (< 0.367 ppm)	Tertile 2 (0.367 ~ 1.102 ppm)	Tertile 3 (> 1.102 ppm)	
N	196	65	65	66	
Season, n (%)					0.375
Spring	90 (45.9)	31 (47.7)	32 (49.2)	27 (40.9)	
Summer	14 (7.1)	6 (9.2)	5 (7.7)	3 (4.5)	
Autumn	49 (25.0)	19 (29.2)	14 (21.5)	16 (24.2)	
Winter	43 (21.9)	9 (13.8)	14 (21.5)	20 (30.3)	
Age, years	74.1 ± 0.52	75.9 ± 1.09	72.7 ± 1.01	74.2 ± 0.94	0.087
Male, n (%)	146 (74.5)	49 (76.6)	45 (69.2)	52 (78.8)	0.474
Smoking status, n (%)					0.788
None	86 (43.9)	29 (44.6)	28 (43.1)	29 (43.9)	
Former	77 (39.3)	26 (40.6)	23 (35.4)	28 (42.4)	
Current	33 (16.8)	10 (15.6)	14 (21.5)	9 (13.6)	
Smoking amount, pack-year	45.0(20.6, 55.0)	38.9(21.3, 43.8)	45.0(35.0, 75.0)	52.3(26.3, 60.0)	** < 0.001**
Comorbidities, n (%)					
Hypertension	92 (46.9)	37 (56.9)	29 (44.6)	26 (39.4)	0.120
Diabetes mellitus	21 (10.7)	5 (7.7)	8 (12.3)	8 (12.1)	0.654
Coronary disease,	25 (12.8)	10 (15.4)	8 (12.3)	7 (10.6)	0.801
Cerebrovascular diseases	23 (11.7)	6 (9.2)	8 (12.3)	9 (13.6)	0.788
Inhaled therapy for COPD, n (%)					
SABA	67 (34.2)	20 (30.8)	21 (32.3)	26 (39.4)	0.562
SAMA	14 (7.1)	2 (3.1)	5 (7.7)	7 (10.6)	0.270
LABA	60 (30.6)	18 (27.7)	25 (38.5)	17 (25.8)	0.429
LAMA	35 (17.9)	11 (17.2)	14 (21.5)	10 (15.4)	0.636
Inhaled corticosteroids	166 (84.7)	57 (87.7)	52 (80.0)	57 (86.4)	0.510
Clinical parameters					
WBC (10^9^/L)	7.3 ± 0.21	5.4 ± 0.16	6.5 ± 0.22	11.2 ± 0.32	** < 0.001**
Neutrophil (10^9^/L)	6.3 ± 0.49	3.5 ± 0.15	5.6 ± 0.92	9.0 ± 0.35	** < 0.001**
Lymphocyte (10^9^/L)	1.5 ± 0.19	1.2 ± 0.08	1.5 ± 0.36	1.2 ± 0.08	0.586
Monocyte (10^9^/L)	0.6 ± 0.02	0.5 ± 0.05	0.6 ± 0.05	0.9 ± 0.05	** < 0.001**
Eosinophil (10^9^/L)	0.08(0.02, 0.18)	0.10(0.05, 0.2)	0.09(0.02, 0.17)	0.07(0.01, 0.16)	0.228
Basophil (10^9^/L)	0.02(0.01, 0.04)	0.02(0.01, 0.04)	0.02(0.01, 0.04)	0.03(0.02, 0.05)	0.190
Uric acid (μmol/L)	301.8 ± 6.78	308.7 ± 13.52	294.7 ± 13.09	286.8 ± 14.41	0.594
Urea nitrogen (mmol/L)	6.7 ± 0.20	6.5 ± 0.33	6.9 ± 0.48	6.5 ± 0.33	0.702
Creatinine (μmol/L)	66.0(53.0, 82.0)	62.0(50.0, 80.0)	68.0(57.0, 86.0)	66.0(55.0, 83.0)	0.429
eGFR (mL/min)	143.1(112.4, 172.5)	141.7 (110.9, 169.2)	124.3(104.1, 163.8)	147.5(137.3, 178.7)	0.116
ALT (U/L)	20.4 ± 1.83	17.0 ± 1.38	17.2 ± 1.01	29.7 ± 6.83	**0.045**
AST (U/L)	24.0 ± 0.97	25.8 ± 2.75	20.8 ± 0.84	26.4 ± 2.30	0.124
IL-6 (pg/mL)	41.6(15.6, 111.5)	19.6(12.2, 39.0)	40.4(18.1, 93.1)	124.7(111.0, 193.6)	** < 0.001**
CRP (mg/L)	33.7(19.0, 60.9)	24.1(15.4, 40.2)	42.2(23.6, 61.0)	84.5(38.9, 150.1)	** < 0.001**
Pulmonary function					
FVC (L)	2.3 ± 0.09	2.6 ± 0.17	2.8 ± 0.18	1.5 ± 0.11	** < 0.001**
FEV1 (L)	1.9 ± 0.08	2.3 ± 0.18	2.2 ± 0.21	1.3 ± 0.08	** < 0.001**
FEV1/FVC (%)	62.5 ± 1.54	67.2 ± 3.89	68.3 ± 3.92	55.6 ± 1.89	**0.002**
FEV1 (%)	60.4 ± 2.69	67.2 ± 3.89	65.4 ± 5.95	40.2 ± 4.49	** < 0.001**

SABA: short-acting beta agonist; SAMA: short-acting muscarinic antagonist; LABA: long-acting beta agonist; LAMA: long-acting muscarinic antagonist; WBCs: white blood cells; eGFR: estimated glomerular filtration rate; ALT: alanine aminotransferase; AST: aspartate aminotransferase; IL-6: interleukin-6; CRP:C-reactive protein; FVC: forced vital capacity; FEV1: forced expiratory volume in one second; FEV1%: predicted FEV1%. Bold values indicate statistical significance

### Progressions in COPD patients within 2 years

**Fig. 1:**
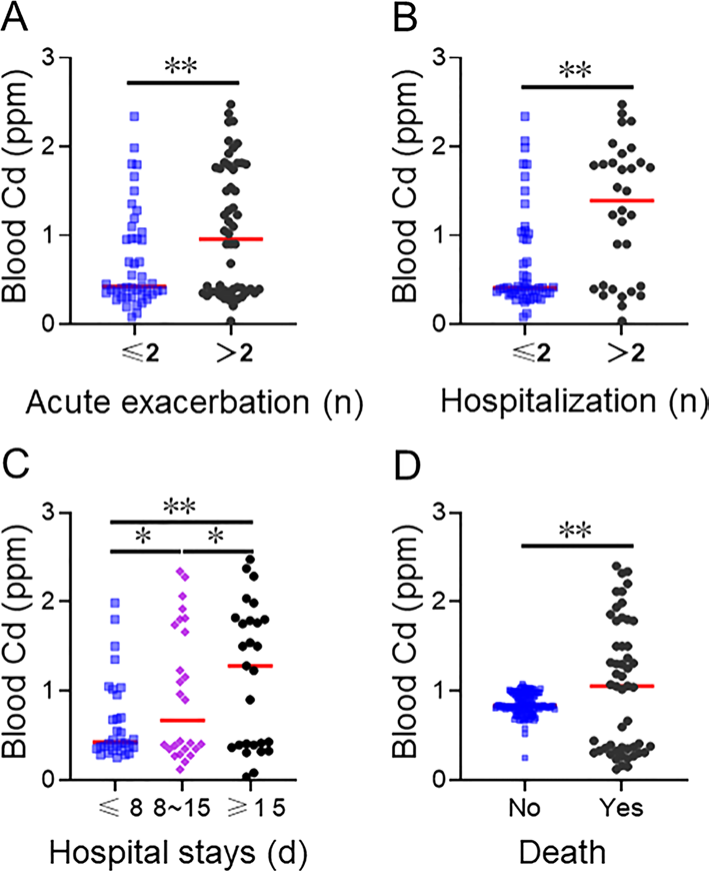
Blood Cd concentrations in COPD patients with different disease progressions. Blood Cd concentration was detected using ICP-MS. The levels of blood Cd concentration were compared in COPD patients with different progressions. **A** Blood Cd concentrations in COPD patients with different acute exacerbations. **B** Blood Cd concentrations in COPD patients with different hospitalizations. **C** Blood Cd concentrations in COPD patients with different hospital stays. **D** Blood Cd concentrations in dead and survived COPD patients. *P < 0.05, **P < 0.01

It was evaluated whether there was a relationship between blood Cd level and COPD progression. According to Table 2, there were glaring distinctions in acute exacerbations, hospitalizations, hospital stays, and death among COPD patients with different blood Cd concentrations. Figure 1 illustrated that as the frequencies of acute exacerbation, hospitalization and duration of hospitalization increased in COPD patients, their blood Cd levels also significantly rose. Furthermore, the levels of Cd in dead COPD patients were higher compared with surviving patients (Fig. 1).

**Table 2:** Relative risk for progressions by tertiles of blood Cd

Prognosis	Tertiles of blood Cd			*P*
	Tertile 1 (< 0.367 ppm)	Tertile2 (0.367 ~ 1.102 ppm)	Tertile 3 (> 1.102 ppm)	
Acute exacerbation (n)	2.7 ± 0.51	2.2 ± 0.45	4.7 ± 0.65	** < 0.001**
Hospitalization (n)	1.3 ± 0.28	1.2 ± 0.23	3.7 ± 0.68	** < 0.001**
Hospital stays (day)	2.6 ± 0.44	2.3 ± 0.36	4.8 ± 0.60	** < 0.001**
Death (n)	15 (23.1)	13 (20.0)	25 (37.9)	**0.048**

Age, sex, smoking status, smoking amount, comorbidities, inhaled therapy for COPD, and pulmonary function indices were adjusted. Bold values indicate statistical significance

### Association of blood Cd concentration with the progression of COPD

**Fig. 2:**
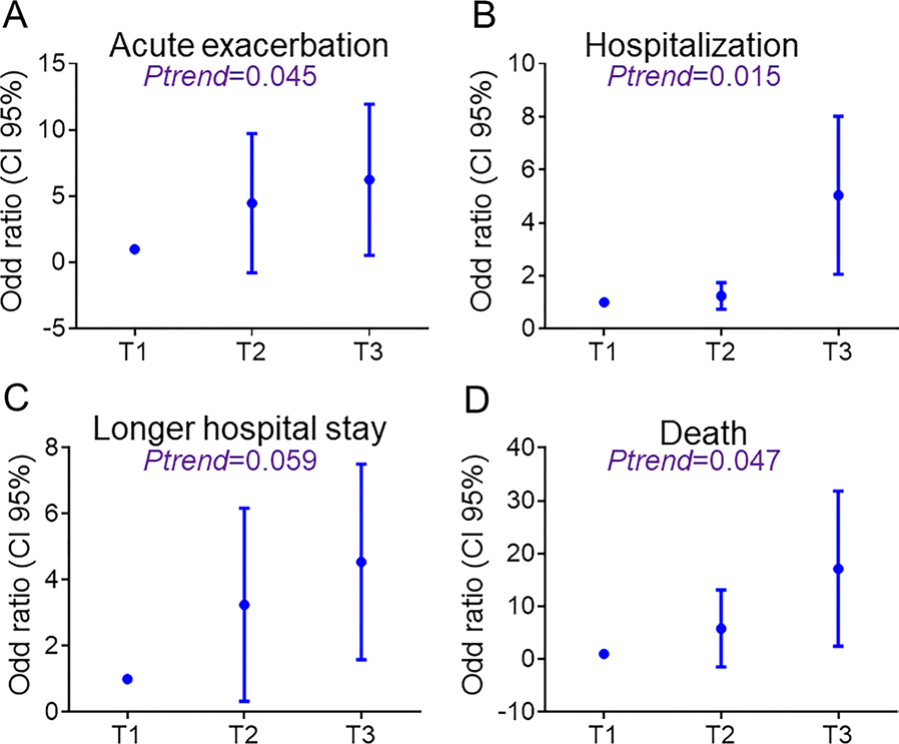
Logistical regression analyses of the association between blood Cd and disease progressions in COPD patients. The odds ratios (ORs) and 95% confidence intervals (CIs) were presented for low blood Cd concentration (T1) compared with medium (T2) and high (T3) blood Cd concentration in COPD patients. **A** The risks of acute exacerbation in COPD patients with different blood Cd concentrations. **B** The risks of hospitalization in COPD patients with different blood Cd concentrations. **C** The risks of longer hospital stay in COPD patients with different blood Cd concentrations. **D** The risks of death in COPD patients with different blood Cd concentrations

Each extra unit of Cd concentration in the univariate binary logistic regression analysis of the entire patient cohort was associated with upward trend in acute exacerbation (OR = 2.238, 95%CI 1.169 ~ 4.288), hospitalization (OR = 1.276, 95%CI 1.134 ~ 1.564), and death (OR = 1.689, 95%CI 1.057 ~ 2.698). Even after taking age, sex, smoking amount, comorbidities, inhaled therapy for COPD, and pulmonary function indices into account, each 1 unit rise in Cd concentration still correlated with increased incidences of acute exacerbation (OR = 2.262, 95%CI: 1.031 ~ 4.243) and death (OR = 2.119, 95%CI: 1.131 ~ 7.113) (Table 3). Interestingly, when adjusting for covariates, the risk of hospitalization did not increase with rising Cd concentration. However, it was worth noting that in both univariate (OR = 3.224, 95%CI 1.382 ~ 7.521) and multivariate (OR = 2.352, 95%CI 1.112 ~ 6.597) analyses, the association between higher Cd concentration and longer hospital stay only existed when the hospital stays were 15 days or more (Table 3). Trend analysis showed that more blood Cd were related to more acute exacerbations, hospitalizations, deaths, and longer hospital stays (Fig. 2).

**Table 3:** The associations of blood Cd with the prognosis in COPD patients

	Univariable (95% CI)	*P*	Multivariable (95% CI) *	*P*
Acute exacerbation (n)				
≤ 2	1	–	1	–
> 2	**2.238 (1.169, 4.288)**	**0.010**	**2.262 (1.031, 4.243)**	**0.044**
Hospitalization (n)				
≤ 2	1	–	1	—
> 2	**1.276 (1.134, 1.564)**	**0.001**	4.053 (0.423, 38.809)	0.254
Hospital stays (d)				
≤ 8	1	–	1	–
8 ~ 15	2.208 (0.937, 5.203)	0.132	0.672 (0.010, 46.030)	0.612
≥ 15	**3.224 (1.382, 7.521)**	**0.005**	**2.352 (1.112, 6.597)**	**0.022**
Death				
No	1	–	1	–
Yes	**1.689 (1.057, 2.698)**	**0.021**	**2.119 (1.131, 7.113)**	**0.045**

^*^Age, sex, smoking amount, comorbidities, inhaled therapy for COPD, and pulmonary function indices were adjusted. Bold values indicate statistical significance

### Stratified analysis for the correlations between blood Cd levels and COPD progressions

In stratified analysis, the correlations between Cd and disease progressions were assessed in participants. There were no meaningful differences in acute exacerbations and hospitalizations in COPD patients with different clinical characteristics (Table 4). In addition, COPD patients with higher age, lower FEV1, and without diabetes mellitus or cerebrovascular diseases more positively affected the association between Cd and hospital stays (Table 4). Moreover, COPD patients with older age, less smoking amount, lower FVC, lower FEV1, and without diabetes mellitus or cerebrovascular diseases had more positive influence on the correlation between Cd and mortality (Table 4).

**Table 4:** Stratified analysis for the associations between blood Cd and prognostic outcomes

Stratification characteristic	Acute exacerbation	Hospitalization	Hospital stays	Death
Age (years)				
≤ 74.0	0.136 (0.013, 1.419)	0.358 (0.065, 1.981)	1.745 (0.366, 8.318)	5.873 (0.529, 65.172)
> 74.0	0.659 (0.058, 7.437)	0.013 (0.002, 73.445)	**1.327 (1.129, 9.819)**	**5.502 (1.169, 25.900)**
*P*_interaction_	0.365	0.254	**0.032**	**0.045**
Sex				
Male	0.307 (0.067, 1.410)	0.324 (0.079, 1.339)	**1.748 (1.024, 5.829)**	**3.358 (1.139, 9.903)**
Female	1.201 (0.325, 2.541)	0.651 (0.205, 1.587)	**1.225 (1.005, 4.638)**	2.025 (0.895, 5.625)
*P*_interaction_	0.958	0.356	0.236	0.524
Smoking amount (n)				
≤ 40.0	4.513 (0.408, 49.919)	0.918 (0.123, 6.832)	**1.809 (1.209, 15.670)**	**9.811 (1.317, 73.0587)**
> 40.0	2.603 (0.306, 22.167)	1.112 (0.658, 2.541)	0.105 (0.003, 3.188)	**6.966 (1.711, 68.224)**
*P*_interaction_	0.524	0.098	0.086	**0.044**
Hypertension				
Yes	1.052 (0.652, 2.365)	1.849 (0.002, 5.628)	5.431 (0.265, 7.958)	3.360 (0.001, 8.954)
No	20.238 (0.333, 128.880)	13.737 (0.423, 446.354)	0.394 (0.043, 3.632)	**5.119 (1.831, 31.526)**
*P*_interaction_	0.632	0.452	0.254	0.254
Diabetes mellitus				
Yes	2.677 (0.652, 4.857)	1.125 (0.658, 4.852)	1.115 (0.777, 5.834)	**2.855 (1.125, 9.562)**
No	2.555 (0.404, 16.172)	1.438 (0.262, 7.9801)	**1.459 (1.289, 6.396)**	**3.660 (1.440, 15.953)**
*P*_interaction_	0.325	0.258	**0.043**	**0.036**
Coronary disease				
Yes	8.315 (0.652, 13.658)	1.258 (0.687, 2.641)	**1.362 (1.112, 3.658)**	1.121 (0.958, 3.521)
No	3.260 (0.433, 24.531)	1.438 (0.923, 1.240)	0.736 (0.156, 3.461)	**3.691 (1.169, 15.679)**
*P*_interaction_	0.154	0.325	0.365	0.859
Cerebrovascular diseases				
Yes	1.251 (0.658, 7.958)	0.658 (0.112, 4.658)	**1.285 (1.058, 5.628)**	**1.652 (1.115, 4.987)**
No	2.911 (0.443, 19.111)	0.696 (0.127, 3.823)	**1.359 (1.009, 6.396)**	**3.710 (1.070, 15.823)**
*P*_interaction_	0.332	0.215	**0.032**	**0.045**
FVC (L)				
≤ 2.15	0.999 (0.733, 2.156)	0.976 (0.265, 1.854)	0.958 (0.365, 1.254)	**2.067 (1.300, 4.652)**
> 2.15	3.627 (0.587, 6.528)	0.989 (0.528, 1.547)	1.654 (0.687, 2.154)	1.339 (0.091, 29.265)
*P*_interaction_	0.069	0.215	0.063	**0.036**
FEV1 (L)				
≤ 1.83	1.254 (0.658, 2.654)	1.125 (0.658, 1.587)	**3.025 (1.115, 7.895)**	**2.635 (1.325, 7.854)**
> 1.83	0.969 (0.658, 2.152)	1.108 (0.778, 2.591)	2.625 (0.879, 3.654)	**1.895 (1.112, 4.628)**
*P*_interaction_	0.138	0.652	**0.025**	**0.030**
FEV1/FVC (%)				
≤ 62.45	1.125 (0.857, 3.254)	1.168 (0.789, 2.635)	1.652 (0.658, 4.658)	**2.025 (1.116, 4.258)**
> 62.45	1.254 (0.758, 2.654)	1.658 (0.857, 3.658)	1.115 (0.878, 3.652)	1.365 (0.879, 3.624)
*P*_interaction_	0.328	0.125	0.205	0.326
Predicted FEV1 (%)				
≤ 54.05	1.259 (0.638, 1.698)	1.326 (0.832, 2.365)	0.788 (0.451, 8.152)	2.630 (0.154, 10.385)
> 54.05	0.994 (0.411, 2.365)	1.111 (0.658, 2.635)	2.081 (0.658, 2.635)	1.366 (0.879, 2.920)
*P*_interaction_	0.268	0.360	0.428	0.365

Models were adjusted for age, gender, smoking amount, comorbidities, inhaled therapy for COPD, and pulmonary function indices. Bold values indicate statistical significance

## Discussion

Cd exposure is fairly common in daily life. It is evident that Cd contamination poses significant health and ecological problems. Furthermore, both the general public and COPD patients are exposed to more environmental Cd when adverse weather conditions are present [37]. The earlier studies have explored the association between Cd exposure and pulmonary function decline in general populations. A population-based study conducted in the United States finds a negative correlation between Cd and FEV1 and FVC in children [3]. Furthermore, a cross-sectional data from another study shows that blood Cd levels are linked to a deterioration of lung function in males [29]. Moreover, other study indicates that blood Cd in individual is linked to decreased FEV1 and FEV1/FVC as well as increased airflow limitation [34, 45]. In addition, our previously cross-sectional studies have found that lower lung function is positively linked to higher blood Cd levels in COPD patients [16, 47]. However, the specific correlation between Cd and the progression of COPD remains uncertain. In this research, we learned that the level of Cd in blood in COPD patients with more acute exacerbations is higher than that with less acute exacerbations. Similarly, blood Cd concentration was increased in COPD patients with more hospitalizations, mortalities, and longer stay in the hospital. Logistic regression analysis indicated that blood Cd concentration elevates the risk of poor progression in COPD patients within 2 years. Therefore, these results suggested that blood Cd concentration is intimately connected with the progression of COPD.

As we all know, many behavioral factors and clinical characteristics may also affect the progression of COPD. Thus, the influences of behavior factors and clinical characteristics on the relationship between Cd and the progression of COPD were explored through stratified analyses. First, we discovered that the link between Cd and progression of COPD is more obvious in COPD patients who are older. The process of aging is linked to the presence of chronic inflammation in the lungs, as well as the alterations of lung structure in COPD patients [6, 14]. Thus, we speculated that COPD patients with older age exhibit higher vulnerability to the effects of Cd exposure. In COPD patients, a reduction in lung function is linked to higher risks of mortality and hospitalization [7, 25, 28]. So, we speculated that patients with poor lung function are more likely to accelerate Cd-associated COPD progression. This is consistent with our conclusion. COPD patients with lower pulmonary function had more positive influence on the association between blood Cd and mortality. Moreover, a cohort study reveals that COPD patients with comorbidities elevate the risks of hospitalization, longer hospital stay, and mortality [19]. According to earlier research, personal PM2.5 exposure has a greater impact on pulmonary function in non-smokers than in smokers. Researchers postulate that smoking may potentially mask the deleterious consequences of exposure to PM2.5, as it has a more pronounced influence on pulmonary function decline compared to PM2.5 exposure [27]. In our study, we learned that COPD sufferers with lower smoking amount and in the absence of comorbidities have more influence on the association between blood Cd concentration and mortality. Maybe comorbidities and higher smoking amount upregulated more mortality compared with environmental Cd exposure in COPD patients.

Mounting data have revealed that inflammation, oxidative stress, epithelial–mesenchymal transition (EMT), autophagy, and apoptosis are involved in the initiation and development of COPD [5, 11, 47]. However, the mechanisms by which Cd induces COPD progression remain unclear. The previous study hints that blood Cd concentration is positively correlated with the levels of inflammatory cytokines among COPD patients [16]. An animal experiment has demonstrated that chronic Cd exposure induces COPD model of mice [42]. Cd exposure obviously elevates airway inflammation and oxidative stress in rats with emphysema [19]. Moreover, a cross-sectional study reveals that blood Cd concentration is positively associated with EMT in lung tissues of COPD patients [2]. In vitro experiments have found that Cd exposure induces EMT in human pulmonary epithelial cells [47]. In vivo experiment also validates that chronic Cd exposure evokes EMT in mice lungs of COPD model [42]. Meanwhile, earlier research suggests that blood Cd concentration is positively associated with the expressions of autophagy and apoptosis in COPD patients [41]. Animal experiment finds Cd exposure can incur autophagy and apoptosis in lungs [39, 46]. In human bronchial epithelial cells, Cd has been shown to cause both autophagy and apoptosis [44]. Therefore, we speculate that Cd may cause COPD through different mechanisms, such as inflammation, oxidative stress, EMT, autophagy, apoptosis, and etc. However, our study was only epidemiological research, the exact mechanisms of Cd-mediated COPD progression can’t be established in the current investigation. More mechanistic researches are needed in the future.

This study had many advantages. First, this investigation revealed the relationship between Cd exposure and the progression of COPD. Second, this study was a multi-center study, with all participants from three separate tertiary hospitals. Third, our follow-up research was carefully evaluated by trained professionals, and data were collected accordingly. However, the research did have some limitations. First, it was unclear how higher Cd affected COPD deterioration, so we need to further explore on how Cd accelerates the progression of COPD. Second, this study's sample size was limited, and the follow-up period was 2 years. Future research should increase the size of sample and lengthen the follow-up time. Third, since this research solely examined the impact of Cd, one of the heavy metals, on the progression of COPD. More heavy metals should be analyzed in future research.

## Conclusions

The comprehensive analysis of this study revealed that blood Cd concentration was associated with the COPD progression. A higher blood Cd level was linked to a worse progression of COPD, including increased risks of acute exacerbation, hospitalization, longer hospital stay, and mortality. This finding highlighted the significance of long-term environmental Cd exposure as a major public health concern. Understanding the impact of Cd exposure on COPD patients can aid in developing targeted interventions to improve patients' outcomes.

## Data Availability

Not applicable.

## Abbreviations

COPD: Chronic obstructive pulmonary disease
Cd: Cadmium
AHCC: The Anhui COPD cohort
GOLD: Global Chronic Obstructive Pulmonary Disease
ICP-MS: Inductively coupled plasma mass spectrometry
FVC: Forced vital capacity
FEV1: Forced expiratory volume in one second
OR: Odds ratios
CI: Confidence intervals
CRP: C-reactive protein
IL-6: Interleukin-6
WBCs: White blood cells
ALT: Alanine aminotransferase
AST: Aspartate aminotransferase
eGFR: Estimated glomerular filtration rate
EMT: Epithelial–mesenchymal transition
